# Dose-dependency and reversibility of radiation-induced injury in cardiac explant-derived cells of mice

**DOI:** 10.1038/srep40959

**Published:** 2017-01-18

**Authors:** Lan Luo, Chen Yan, Yoshishige Urata, Al Shaimaa Hasan, Shinji Goto, Chang-Ying Guo, Shouhua Zhang, Tao-Sheng Li

**Affiliations:** 1 Department of Stem Cell Biology, Nagasaki University Graduate School of Biomedical Sciences, 1-12-4 Sakamoto, Nagasaki 852-8523, Japan; 2 Jiangxi Cancer Hospital, Nanchang, Jiangxi 330029, PR China; 3 Department of General Surgery, Jiangxi Provincial Children’s Hospital, Nanchang, Jiangxi Province, 330006, PR China

## Abstract

We evaluated the dose-dependency and reversibility of radiation-induced injury in cardiac explant-derived cells (CDCs), a mixed cell population grown from heart tissues. Adult C57BL/6 mice were exposed to 0, 10, 50 and 250 mGy γ-rays for 7 days and atrial tissues were collected for experiments 24 hours after last exposure. The number of CDCs was significantly decreased by daily exposure to over 250 mGy. Interestingly, daily exposure to over 50 mGy significantly decreased the c-kit expression and telomerase activity, increased 53BP1 foci in the nuclei of CDCs. However, CD90 expression and growth factors production in CDCs were not significantly changed even after daily exposure to 250 mGy. We further evaluated the reversibility of radiation-induced injury in CDCs at 1 week and 3 weeks after a single exposure to 3 Gy γ-rays. The number and growth factors production of CDCs were soon recovered at 1 week. However, the increased expression of CD90 were retained at 1 week, but recovered at 3 weeks. Moreover, the decreased expression of c-kit, impaired telomerase activity, and increased 53BP1 foci were poorly recovered even at 3 weeks. These data may help us to find the most sensitive and reliable bio-parameter(s) for evaluating radiation-induced injury in CDCs.

Radiation exposures are generally classified as high (above 5 Gy), moderate (0.5~5 Gy) and low doses (below 0.5 Gy)^1^. Epidemiological studies on the atomic bomb survivors of Hiroshima and Nagasaki^2^,^3^, workers from the Mayak nuclear facility in the Russian Federation^4^,^5^,^6^, and the Chernobyl liquidators^7^ have clearly suggested that high dose ionizing radiation increase the cardiovascular disease (CVD) risk^8^,^9^. However, the CVD risks at doses below 0.5 Gy has been weakly evidenced due to the low statistical power and uncertainty in dose assessment^10^,^11^. Therefore, it keeps controversial whether low and moderate doses of ionizing radiation exposure also contribute to future CVD risk^12^. In respect of medical use of radiation for diagnosis, occupational and environmental radiation exposure, there is a strong need to understand the radiation-induced CVD risk at doses less than 0.5 Gy.

The tissue-specific stem/progenitor cells are well known to play critical roles in maintaining the homeostasis of tissues/organs under physiological condition and for repairing after pathological damages. Many studies have also posed the damaged stem cells as the initiators of radiation-induced carcinogenesis^13^,^14^. Thus, radiation-induced injury in stem cells may closely associate with future cancer and non-cancer risks^15^. Consistent with previous study, we have successfully expanded cells from the “explants” of heart tissues of animals, and the cardiac explant-derived cells (CDCs) revealed a mixed cell population, which extensively expressed with mesenchymal marker of CD105 but also barely expressed with common stem cell marker of c-kit^16^. Recently, we have demonstrated that whole-body radiation exposure to a moderate dose of 3 Gy γ-rays significantly induced injury to CDCs, including the decreased cell outgrowth, the changes of cell phenotypes, the decreased telomerase activity, the increased DNA damage and the impaired production of growth factors^17^. However, it is asked to confirm the most sensitive and reliable bio-parameter(s) for detecting radiation-induced injury in CDCs. Also, it is of great interest to know whether the radiation-induced injury in CDCs will be temporary or permanent.

In this study, we first daily exposed mice to different doses of γ-rays (0 to 250 mGy/day) for 7 days, and then detected the dose-dependency of radiation-induced injury in CDCs by various bio-parameters. Alternatively, the reversibility of radiation-induced injury in CDCs was investigated at 1 and 3 weeks after a single exposure of mice to 3 Gy γ-rays. Our data showed the differences on the sensitivity and reversibility among bio-parameter(s) for evaluating radiation-induced injury in CDCs.

## Results

### Cell phenotypes of CDCs

**Figure 1:**
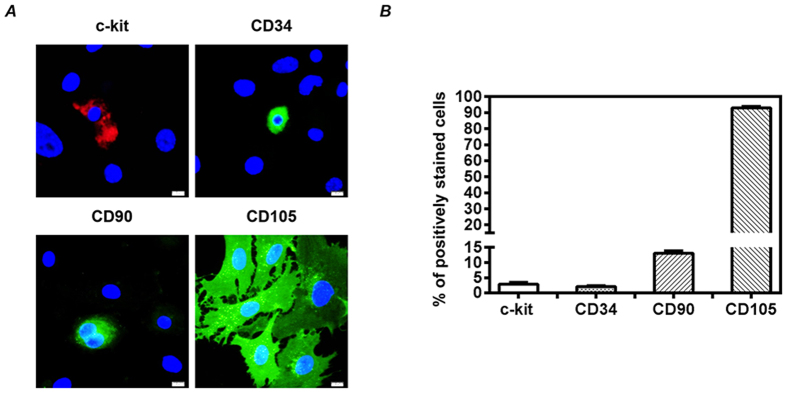
Cell phenotypes of cardiac explant-derived cells (CDCs). Healthy mouse atrial tissues were collected for expansion of CDCs. To characterize the cell phenotypes of CDCs, we did immunostaining of twice-passaged CDCs with c-kit, CD34, CD90 and CD105. (**A**) Representative images were shown, scale bar: 10 μm. (**B**) Quantitative data were obtained by counting the positively stained cells from 20 randomly selected fields. Values are the mean ± SD (n = 3).

To characterize the cell phenotypes of cardiac explant-derived cells, immunostaining with c-kit, CD34, CD90 and CD105 were performed in twice-passaged CDCs expanded from atrial tissues of healthy mice (Fig. 1A). The majority of CDCs expressed CD105 (93.00%), and a few of CDCs expressed c-kit (2.92%), CD34 (2.10%) and CD90 (13.08%) (Fig. 1B). Based on these findings, CDCs appeared to be a mixed cell population with extensive expression of CD105.

### Dose-dependency of radiation-induced injury in CDCs

**Figure 2:**
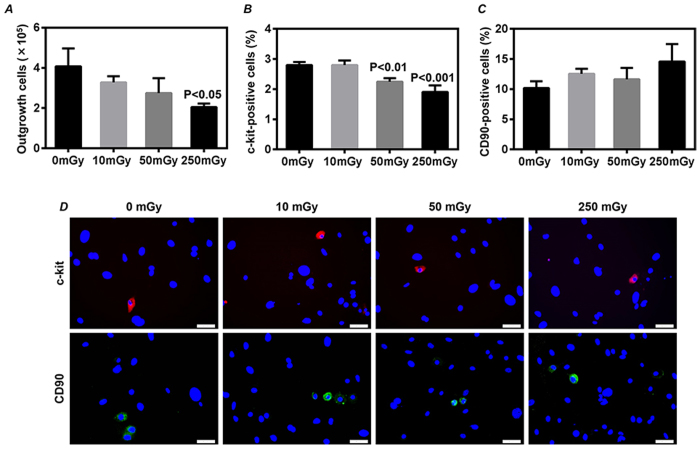
The number and phenotypic characterization of cardiac explant-derived cells (CDCs). The mouse atrial tissue was collected for expansion of CDCs after daily exposures to 0, 10, 50 and 250 mGy γ-rays for 7 days. (**A**) CDCs were harvested at day 14, and the number of total collected CDCs from each mouse was directly counted. The phenotypic characterization was determined by immunostaining on the expression of c-kit (**B**) and CD90 (**C**) in twice-passaged CDCs. Quantitative data were obtained by counting the positively stained cells from 20 randomly selected fields. Representative images of immunostaining were shown (**D**). Values are the mean ± SD (n = 3). Scale bar: 50 μm.

All mice survived from the daily radiation exposure to 0~250 mGy γ-rays for 7 days. Although the well-trained skill with a defined protocol, daily exposed to 250 mGy for 7 days significantly decreased the number of CDCs that expanded from atrial tissues of mice (*P* < 0.05 *vs.* 0 mGy, Fig. 2A). Moreover, by immunostaining with the common stem cell marker of c-kit, we found the expression of c-kit in CDCs was significantly decreased after daily exposure to over 50 mGy for 7 days (*P* < 0.01 *vs.* 0 mGy, Fig. 2B). However, the expression of CD90, a mesenchymal marker in CDCs was not significantly changed after exposures to a range of 0~250 mGy for 7 days (Fig. 2C).

**Figure 3:**
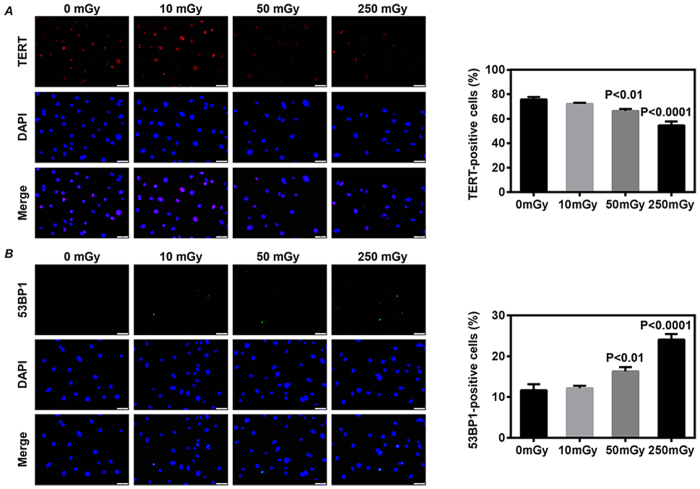
Telomerase activity and DNA damage of cardiac explant-derived cells (CDCs). CDCs were expanded from mouse after daily exposures to 0, 10, 50 and 250 mGy γ-rays for 7 days. The telomerase activity (**A**) and DNA damage (**B**) in the twice-passaged CDCs were evaluated by immunostaining on the expression of telomerase reverse transcriptase (TERT) and 53BP1, respectively. Representative images were shown (left images). Quantitative data (right bar graphs) were obtained by counting the positively stained cells from 20 randomly selected fields. Values are the mean ± SD (n = 3). Scale bar: 50 μm.

The cell senescence of CDCs was evaluated by immunostaining with telomerase reverse transcriptase (TERT). The expression of TERT was significantly decreased in the CDCs from mice daily exposed to over 50 mGy γ-rays for 7 days (*P* < 0.01 *vs.* 0 mGy, Fig. 3A). The percentage of cells with 53BP1 foci, a marker for DNA damage, was also significantly increased in the CDCs from mice daily exposed to over 50 mGy γ-rays for 7 days (*P* < 0.01 *vs.* 0 mGy, Fig. 3B).

**Figure 4:**
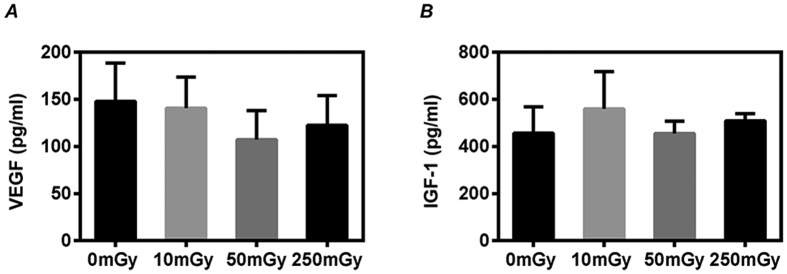
Growth factors production of cardiac explant-derived cells (CDCs). CDCs were expanded from mouse after daily exposures to 0, 10, 50 and 250 mGy γ-rays for 7 days. The supernatants from the twice-passaged CDCs were collected for measuring the concentration of VEGF (**A**) and IGF-1 (**B**) by ELISA. Values are the mean ± SD (n = 3).

We further examined the production of VEGF and IGF-1, two important beneficial factors for myocardial repair. The production of VEGF (Fig. 4A) and IGF-1 (Fig. 4B) from CDCs was not significantly changed among all groups received daily exposure with 0~250 mGy γ-rays for 7 days.

### Reversibility of radiation-induced injury in CDCs

**Figure 5:**
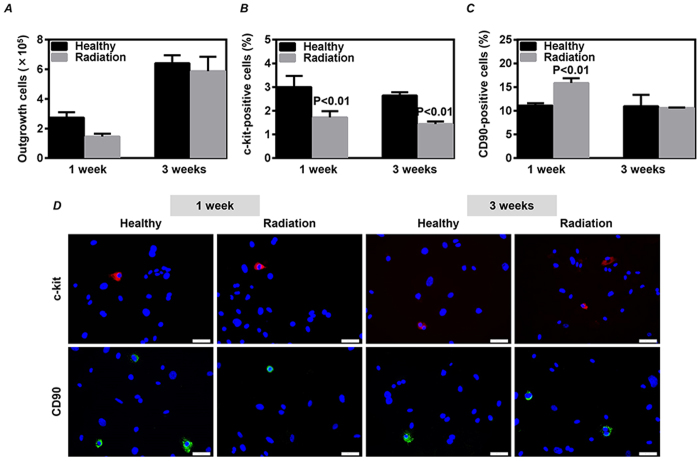
Recovery on the number and phenotypic characterization of cardiac explant-derived cells (CDCs) after radiation exposure. Mouse atrial tissue was collected for the expansion of CDCs at 1 and 3 weeks after a single exposure to 3 Gy γ-rays. (**A**) CDCs were harvested at day 14, and the number of total collected CDCs from each mouse was directly counted. The phenotypic characterization was determined by immunostaining on the expression of c-kit (**B**) and CD90 (**C**) in the twice-passaged CDCs. Quantitative data were obtained by counting positively stained cells from 20 randomly selected fields. Representative images of immunostaining were shown (**D**). Values are the mean ± SD (n = 3). Scale bar: 50 μm.

To know whether the radiation-induced injury in CDCs would be reversible, we exposed mice to 3 Gy γ-rays and collected the heart atrial tissue for *ex vivo* expansion of CDCs 1 and 3 weeks later. Comparing to the healthy mice, the number of CDCs harvested from the atrial tissue of irradiated mice returned to a comparable level between groups at 1 and 3 weeks of follow-up after radiation (Fig. 5A).

The expression of c-kit was significantly decreased in CDCs from the irradiated mice at 1 week (*P* < 0.01 *vs.* Healthy, Fig. 5B) and 3 weeks of follow-up after radiation (*P* < 0.01 *vs.* Healthy, Fig. 5B). Although the expression of CD90 was significantly increased in CDCs from the irradiated mice at 1 week (*P* < 0.01 *vs.* Healthy, Fig. 5C), it was observed a comparable level between groups at 3 weeks of follow-up after radiation (Fig. 5C).

**Figure 6:**
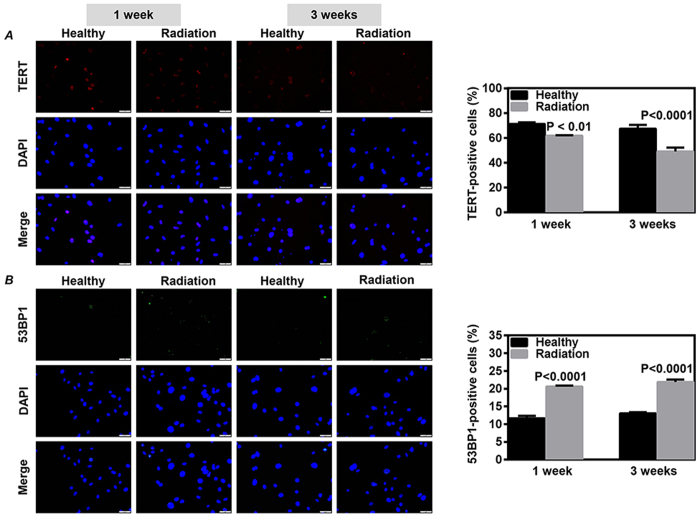
Recovery on the telomerase activity and DNA damage of cardiac explant-derived cells (CDCs) after radiation exposure. CDCs were expanded from mouse at 1 and 3 weeks after a single exposure to 3 Gy γ-rays. The telomerase activity (**A**) and DNA damage (**B**) in the twice-passaged CDCs were evaluated by immunostaining on the expression of telomerase reverse transcriptase (TERT) and 53BP1, respectively. Representative images were shown (left images). Quantitative data (right bar graphs) were obtained by counting positively stained cells from 20 randomly selected fields. Values are the mean ± SD (n = 3). Scale bar: 50 μm.

We also examined the cell senescence and DNA damage in CDCs. The expression of TERT was significantly lower in CDCs from irradiated mice than that of healthy mice at 1 week (*P* < 0.01 *vs.* Healthy, Fig. 6A) and 3 weeks (*P* < 0.0001 *vs.* Healthy, Fig. 6A) of follow-up after radiation. The percentage of cells with 53BP1 foci was also observed to be significantly higher in CDCs from irradiated mice than that of healthy mice at 1 week (*P* < 0.0001 *vs*. Healthy, Fig. 6B) and 3 weeks of follow-up after radiation (*P* < 0.0001 *vs*. Healthy, Fig. 6B).

**Figure 7:**
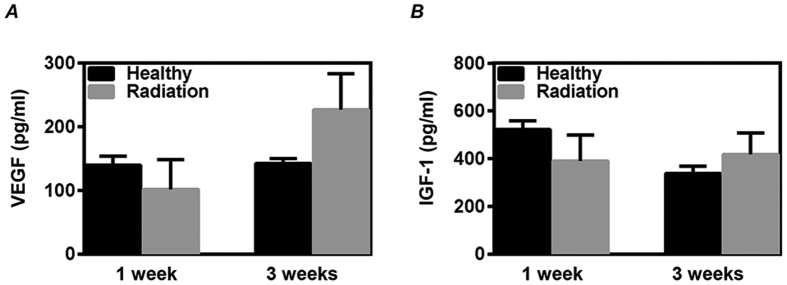
Recovery on the production of growth factors from cardiac explant-derived cells (CDCs) after radiation exposure. CDCs were expanded from mouse at 1 and 3 weeks after a single exposure to 3 Gy γ-rays. The supernatants from the twice-passaged CDCs were collected for measuring the concentration of VEGF (**A**) and IGF-1 (**B**) by ELISA. Values are the mean ± SD (n = 3).

We further examined the production of VEGF and IGF-1 from CDCs *in vitro*. Compared with CDCs from healthy mice, CDCs from the irradiated mice showed no significant change in the production of VEGF and IGF-1 at 1 and 3 weeks of follow-up after radiation, suggesting the completely recovery on VEGF and IGF-1 production of CDCs (Fig. 7).

## Discussion

This study was designed to examine the dose-dependency and reversibility of radiation-induced injury in cardiac explant-derived cells, a mixed population of cardiac stem cells and supporting cells grown from heart tissues. By daily exposure of healthy mice to 0~250 mGy γ-rays for 7 days, we demonstrated that radiation exposure declined the number and c-kit expression, and induced cell senescence and DNA damage of CDCs in a dose-dependent manner. After a single exposure to 3 Gy γ-rays, the decreased number of CDCs was recovered in 1 week. However, the decreased c-kit expression, the impaired telomerase activity, and the increased DNA damage in CDCs were poorly recovered even at 3 weeks after a single exposure to 3 Gy γ-rays. Otherwise, radiation exposure had limitedly affected the property of CDCs for the production of VEGF and IGF-1, two important growth factors that well known to be beneficial of myocardial regeneration/repair^18^,^19^.

Up to present, the evidence on potential cardiovascular disease risk after low dose radiation exposure is still indecisive, and the underlying cellular and molecular mechanisms remain unclear^10^. We are interested to uncover the radiation-induced cardiovascular disease risk by focusing the injury of resident cardiac explant-derived cells in heart. Using a panel of bio-parameters, we have very recently demonstrated that a single exposure to 3 Gy γ-rays can impair cardiac explant-derived cells in quantity and quality^17^. To further identify the most sensitive and reliable bio-parameter(s) for future studies, we herein tested the dose-dependency and reversibility of radiation-induced injury in CDCs.

By daily exposure of healthy mice to 0~250 mGy γ-rays for 7 days, the dose-dependency on radiation-induced injury of CDCs was clearly evidenced by the decreased c-kit expression, the impaired telomerase activity, and the increased formation of 53BP1 foci in CDCs. However, other bio-parameters, such as the total cell number of CDCs and their production of beneficial growth factors did not show dose-dependent change after radiation exposure. Agreed well with the results from dose-dependency experiments, the c-kit expression, the telomerase activity, and the formation of 53BP1 foci in CDCs were poorly recovered even at 3 weeks after radiation exposure. However, other bio-parameters, such as the total cell number and growth factors production were completely restored as early as 1 week after a single exposure to 3 Gy γ-rays, suggesting that total cell number and growth factors production were not likely to be the sensitive/reliable bio-parameters for detecting radiation-induced injury in CDCs.

Respectively, the telomerase activity and the 53BP1 foci formation are popularly used to indicate the senescence and DNA damage of cells. The shortening of telomere occurs with organismal aging, and it is accelerated in the pathobiology of human disease^20^. Recent studies have also suggested that telomere length and telomerase activity can directly influence the ability of stem cells to regenerate tissues^21^,^22^. Therefore, the radiation-induced decrease of telomerase activity in CDCs may lead to an insufficient regeneration/repair, which finally results in the increased cardiovascular disease risk. Consistent with our finding, it has been found that radiation exposure to moderate and low doses can induce DNA damage in hematopoietic stem/progenitor cells^15^. The accumulated DNA damage is well known to induce cell cycle arrest and associate with carcinogenesis.

According to our data, daily exposure to over 50 mGy γ-rays for 7 days significantly decreased the telomerase activity and increased the formation of 53BP1 foci in CDCs. Moreover, the radiation-induced senescence and DNA damage in CDCs were not able to recover within 3 weeks follow-up after a single exposure to 3 γ-rays. Therefore, telomerase activity and the 53BP1 foci formation seem to be the reliable and sensitive bio-parameters for detecting radiation-induced injury of CDCs.

There are several limitations in this study. First, we only investigated the radiation-induced damages in cardiac explant-derived cells, a mixed cell population, because the cardiac explant-derived cells can be easily and stably expanded from cardiac explants and have been clinically tested for myocardial repair^23^,^24^. It will be also interesting to know the radiation effect specially on the small fraction of c-kit-positive cells in heart^25^,^26^. However, it is technically difficult for us to get enough number of c-kit-positive cells for experiments because the expression of c-kit is extremely low in the heart tissue of mouse and may loss during *ex vivo* expansion^27^. Second, radiation exposure was done at a high dose rate (>12 Gy per hour) in our study. As the dose rate has been largely taken into consideration on radiation-induced cancer risk^28^, it will be of interesting to evaluate the radiation-induced injury in CDCs with a low dose rate exposure (<5 mGy per hour). Third, the reversibility of radiation-induced injury in CDCs was only investigated at 1 and 3 weeks after a single exposure to 3 Gy γ-rays due to the very limited foundation and man-power. It is of great interest to test the reversibility with different doses in the future. Fourth, we did not clearly confirm the lowest dose range for radiation-induced injury in CDCs. Last, but not the least, we didn’t unravel the exact mechanisms on radiation-induced injury of CDCs at gene or protein levels, especially about the cell senescence and DNA damage. Further experiments about radiation-induced injury of CDCs are barely needed with even longer-term follow-up after the exposure to wide dose range.

In all, data from this study provided board information about the sensitivity and reversibility of radiation-induced injury in CDCs. What’s more, the identification of sensitive and reliable bio-parameter(s) for detecting radiation-induced injury in CDCs may finally help us to predict the future radiation-induced cardiovascular disease risk.

## Methods

### Animals

Adult (10–14-week-old) male C57BL/6 mice (CLEA Japan, Inc.) were used for the experiments. This study was approved by the Institutional Animal Care and Use Committee of Nagasaki University (No. 1108120943-8), and all animal procedures were performed in accordance with the institutional and national guidelines.

### Radiation exposures

To evaluate the dose-dependency of radiation-induced injury in CDCs, using a PS-3100SB γ-ray irradiation system with a Cs source (Pony Industry Co., Ltd. Osaka, Japan), we exposed the mice to 0, 10, 50 and 250 mGy γ-rays daily in succession for 7 days (with accumulative doses of 0, 70, 350 and 1750 mGy) at a dose rate of 0.886 Gy/min, respectively^29^. Then mice were euthanized by severing the aorta under general anesthesia with intraperitoneal injection of 160 mg/kg pentobarbital 24 hours after irradiation. The heart was quickly injected with 5 ml cold cardioplegic solution (Mochida Pharmaceutical Co., LTD.), and the atrial tissue was collected for *ex vivo* expansion of CDCs as described below^17^. To evaluate the reversibility of radiation-induced injury in CDCs, mice were received a single exposure to 0 or 3 Gy γ-rays, and then sacrificed for experiment 1 and 3 weeks later.

### *Ex vivo* expansion of CDCs

CDCs were expanded using methods similar to those previously described^17^. In brief, mice atrial tissues were minced into small fragments and cultured as “explants” on 6-cm fibronectin (15 μg/ml) coated culture dishes. Within 1 week, stromal-like flat cells and phase-bright round cells grown from the tissue fragments and became confluent at approximately 2 weeks. The outgrowth of CDCs was harvested using 0.25% trypsin (Gibco) at 2 weeks, counted using a Nucleo Counter cell-counting device (Chemotetec A/S, Denmark), and then passaged for cell expansion. Twice-passaged CDCs were used for the following experiments as indicated. All cells were cultured in a 5% CO_2_ incubator at 37 °C using IMDM basic medium (Gibco) supplemented with 10% fetal bovine serum (HyClone), 100 units/ml penicillin G and 10 μg/ml streptomycin (WAKO, Japan).

### Characterization of CDCs

The expression levels of c-kit, CD34, CD90 and CD105 in CDCs were estimated by immunostaining^17^. In brief, twice-passaged CDCs (1 × 10^4^/well) were cultured in 8-well chamber culture slides (Lab-Tek, Thermo Scientific Nunc) coated with 15 μg/ml fibronectin. The cells were fixed in 4% paraformaldehyde for 10 minutes after 3 days of culture. After blocking, the cells were incubated with PE-conjugated rat anti-mouse c-kit antibody (eBioscience), FITC-conjugated rat anti-mouse CD34 antibody (eBioscience), or with rat anti-mouse CD90 monoclonal antibody (Abcam) and rat anti-mouse CD105 monoclonal antibody (Abcam) followed by donkey anti-rat Alexa Flour 488-conjugated secondary antibody. Nuclei were stained with 4′,6-diamidino-2-phenylin-dole (DAPI), and the positively stained cells were counted under a fluorescent microscope with 200-fold magnification. Twenty fields per slide were randomly selected for quantitative counting.

### Evaluation of cell senescence and DNA damage

To evaluate the cell senescence and DNA damage, twice-passaged CDCs (1 × 10^4^/well) were seeded on 8-well chamber culture slides as mentioned above^17^. After 3 days of culture, the cells were fixed in 4% paraformaldehyde for 10 min. After blocking, the cells were incubated with rabbit polyclonal antibodies against mouse telomerase reverse transcriptase-C-terminal or 53BP1 (Abcam). Positive staining was detected by appropriate second antibodies conjugated with Alexa fluorochromes. The nuclei were stained with DAPI, and the positively stained cells were counted under a fluorescent microscope with 200-fold magnification. Twenty fields per slide were randomly selected for quantitative counting.

### ELISA

Conditioned media was collected from twice-passaged CDCs 3 days after culture in 8-well chamber culture slides^17^. A mouse VEGF ELISA kit and a mouse/rat IGF-1 ELISA kit (R&D Systems) were used to measure the levels of VEGF and IGF-1 in the conditioned media^30^.

### Statistical analysis

All of the results are presented as the mean ±SD. The statistical significance was determined by one-way ANOVA and followed by Turkey’s multiple comparisons test (GraphPad Prism). Differences were considered significant when *P* < 0.05.

## Additional Information

**How to cite this article**: Luo, L. *et al*. Dose-dependency and reversibility of radiation-induced injury in cardiac explant-derived cells of mice. *Sci. Rep.* **7**, 40959; doi: 10.1038/srep40959 (2017).

**Publisher's note:** Springer Nature remains neutral with regard to jurisdictional claims in published maps and institutional affiliations.
